# Community Structure and Influencing Factors of Macro-Benthos in Bottom-Seeded Marine Pastures: A Case Study of Caofeidian, China

**DOI:** 10.3390/biology14070901

**Published:** 2025-07-21

**Authors:** Xiangping Xue, Long Yun, Zhaohui Sun, Jiangwei Zan, Xinjing Xu, Xia Liu, Song Gao, Guangyu Wang, Mingshuai Liu, Fei Si

**Affiliations:** 1 Hebei Key Laboratory of the Bohai Sea Fish Germplasm Resources Conservation and Utilization, Beidaihe Central Experiment Station, Chinese Academy of Fishery Sciences, Qinhuangdao 066100, China; xuexp@bces.ac.cn (X.X.); sunzh@bces.ac.cn (Z.S.); zanjw@bces.ac.cn (J.Z.); xuxj@bces.ac.cn (X.X.); liux@bces.ac.cn (X.L.); gaos@bces.ac.cn (S.G.); 2 Bohai Sea Fishery Research Center, Chinese Academy of Fishery Sciences, Qinhuangdao 066100, China; 3 State Key Laboratory Incubation Base for Conservation and Utilization of Bio-Resource in Tarim Basin, College of Life Sciences and Technology, Tarim Research Center of Rare Fishes, Tarim University, Alar 843300, China; 10757232147@stumail.taru.edu.cn; 4 National Shrimp and Crab Industry Technology System Tangshan Comprehensive Test Station, Tangshan 063299, China; 15032991002@163.com; 5 Tangshan Caofeidian Agricultural Development Group Co., Ltd., Tangshan 063200, China; 13733256085@163.com

**Keywords:** marine pastures, benthic organisms, community structure, environmental factors

## Abstract

To accurately assess the water quality, ecosystem status, distribution of large benthic organisms, and ecological restoration under human intervention, an analysis of benthic organisms on Caofeidian in September and November 2023 and January and May of the following year was conducted in this work. By performing CCA (canonical correspondence analysis) and cluster and correlation coefficient (Pearson) analyses, the temporal variation characteristics of benthic abundance, dominant species, community structure and biodiversity were analyzed. A total of 79 species of macro-benthic animals were found in four months, including 32 species of polychaetes, cnidarians, 1 species of Nemertean, 19 species of crustaceans, and 24 species of molluscs. The use of conventional grab-type mud collectors revealed that the *Musculus senhousei* dominated the survey (*Y* > 0.02). While only a small number of *Ruditapes philippinarum* were collected from bottom-dwelling species, a certain number of bottom-dwelling species (*Ruditapes philippinarum* and *Scapharca subcrenata*) were also collected during the trawl survey. Additionally, a significant population of *Rapana venosa* was found in the area. It is speculated that the dual effects of predation and competition are likely the primary reasons for the relatively low abundance of bottom-dwelling species. The density and biomass of macro-benthos were consistent over time, which was the highest in May, the second highest in January, and the lowest in September and November. The main environmental factors affecting the large benthic communities in the surveyed sea areas were pH, DO, NO_2_^−^-N, T, SAL and PO_4_^3−^-P. Combined with historical data, it was found that although the environmental condition in the Caofeidian sea area has improved, the *Musculus senhousei* has been dominant. In addition, the abundance of other species is much less than that of the *Musculus senhousei*, and the diversity of the benthic community is still reduced. Our work provides valuable data support for the management and improvement of bottom Marine pasture and promotes the transformation of Marine resources from resource plunder to a sustainable resource.

## Simple Summary

Benthic organism surveys were conducted at six stations in a bottom-seeding marine ranch in Caofeidian over four months. A total of 79 species of benthic animals were captured. Among them, there were 32 species of polychaetes, 1 species each of cnidarians, echinoderms, brachiopoda and nemerteans, 19 species of crustaceans, and 24 species of molluscs. The main dominant species in the macro-benthic community were mostly small bivalves and crabs and shrimps that live a burrowing lifestyle. In May, small bivalves became the absolutely dominant species. The average values of H′ in each month of the bottom-seeding marine ranch in Caofeidian showed that the average values in January and November were relatively high, while the average values in May and September were relatively low. The Pearson correlation coefficient and CCA indicated that DO, pH, COD, NO_3_^−^-N, NO_2_^−^-N, PO_4_^3−^-P, and NH_3_-N were the key water environmental factors affecting the density changes in macro-benthos in the bottom-seeding marine ranch in Caofeidian.

## 1. Introduction

Driven by strategies focused on global food security and the blue economy, the mariculture-oriented development model plays a crucial role in the marine economy and related industries [1,2]. This trend is particularly apparent in China. In 2023, the value of China’s marine aquaculture reached CNY 488.548 billion, accounting for 30.62% of the total fishery output. With a yearly production of 23.956 million tonnes, China firmly maintains its position as the world’s largest producer, underscoring the strategic importance of marine aquaculture in the national food supply system. However, the conventional single-species intensive farming model, which involves excessive feeding, leads to eutrophication, degrades wildlife habitats and presents a continuous threat to wild fishery resources. The Food and Agriculture Organization (FAO) of the United Nations predicted that global aquatic product consumption will increase by over 30% by 2050. Marine ranching is viewed as a critical solution to compensate for this predicted decline in wild fishery resources.

As semi-closed ecosystems created through artificial intervention, marine ranches have emerged as sustainable development models that balance ecological protection and resource enhancement [3]. This model, defined as an ‘artificially regulated marine biological resource conservation and enhancement system’, produces a comprehensive balance between ecological protection and resource development via the collaboration of multiple species, habitat creation and the utilization of three-dimensional space. Among various types of marine ranches, the bottom-seeding type is a specialized model focusing on enhancing benthic shellfish and high-quality seabed species [4]. This model is based on scientific spatial planning and quality grading systems, integrated with rotational harvesting agreements, to ensure the sustainable application of resources [5]. This management mechanism not only considerably boosts the sustainability of biological resources and biodiversity protection but also delivers substantial ecological, economic and social benefits. For example, the Tangshan Port Development Zone has implemented a novel ‘company + cooperative + fisherman’ marine ranch win–win model, providing holistic services to nearly 300 fishing boats and creating employment for over 6000 fishers.

In the coastal waters of Tangshan Caofeidian Ecological City, the sandy seabed and excellent water quality support a rich array of marine life, providing a natural foundation for the establishment of bottom-seeded marine ranches. This ecosystem, constructed through seabed substrate improvement and artificial seeding, not only bolsters the sustainable growth of bivalves, sea cucumbers and other bottom-dwelling organisms but also holds strategic value in enhancing marine carbon sequestration and advancing the green transformation of fisheries. It serves as a model for the coordinated development of Bohai Sea ecological protection and resource exploitation. By improving seabed sediment and creating a composite bottom habitat, this method provides attachment and resting spaces for bivalves, sea cucumbers and other bottom-dwelling organisms, championing the coexistence of multi-trophic level organisms. This approach utilizes a multi-species farming model, which prevents ecological imbalance, assists in restoring degraded seabed ecosystems, rebuilds habitats to attract endangered species, reduces reliance on wild populations, and decreases pressure on wild seedling harvesting, effectively protecting the biodiversity of bottom-dwelling organisms [6,7].

Benthic organisms, defined as communities of organisms that inhabit the substrates or sediment–water interfaces in marine and freshwater ecosystems, are a vital component of aquatic ecosystems [8,9]. These organisms are critical in the biogeochemical cycles and energy transfer within aquatic food webs. Based on five vital biological attributes, benthic organisms have been identified as important biological indicators for monitoring aquatic environments. Notably, they exhibit high environmental sensitivity to ecological disturbances, limited mobility with a narrow dispersal range, distinct taxonomic features, longer life cycles and the unique ability to retain specific ecological memories of their habitats as environmental archives. In 2000, Borja et al. introduced the AMBI (AZTI Marine Biodiversity Index), which measures the disturbance induced by human activities by quantifying the ratio of sensitive to tolerant species [10]. These biological characteristics enable a broad range of applications in biological monitoring projects and water quality assessment programs [11,12,13]. In recent years, large benthic organisms have become crucial biological indicators for assessing anthropogenic disturbances and are extensively leveraged in the environmental monitoring of estuarine and coastal ecosystems [14,15,16,17].

However, most existing studies concentrate on the responses and interactions of benthic organisms in natural marine environments to sedimentary conditions. Still, there is a paucity of research on the cascading effects of ‘human intervention–physical and chemical properties of the water environment–community structure’ in bottom-seeding activities. Liang Miao et al.’s investigation of the Caofeidian Marine Area also revealed that the interactions between bottom-seeded species and large benthic organisms, as well as their impact on the water environment, remain a research gap [18]. To address this issue effectively, this study systematically investigates (1) the structural characteristics of large benthic organism communities; (2) their interactions with environmental factors; and (3) the determination of key influencing variables. The findings provide a theoretical foundation for restoring large benthic ecosystems and protecting aquatic biodiversity and also offer a critical dataset for the comprehensive assessment of the ecological status of bottom-seeding marine ranch systems.

## 2. Materials and Methods

### 2.1. Sample and Water Quality Data Acquisition

**Figure 1:**
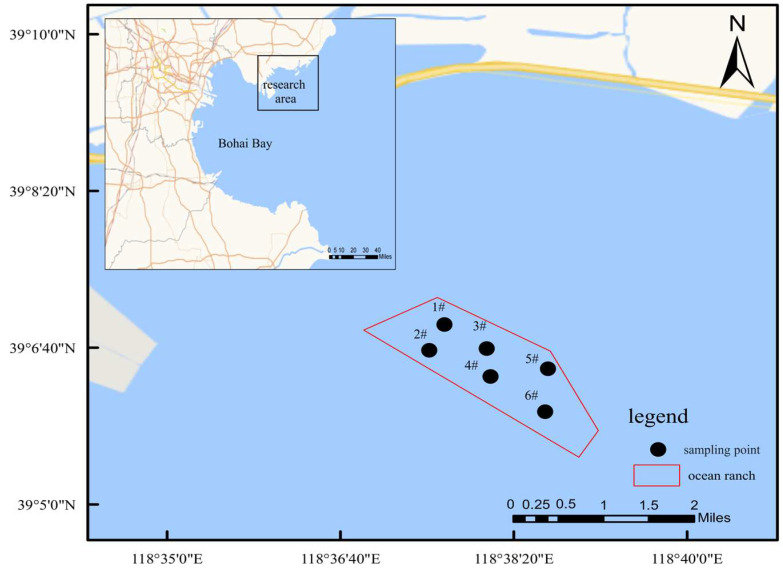
Sampling map of macro-benthic animals in the study area. Note: In the legend, 1#–6# indicate sampling stations 1–6.

The study’s bottom-seeding marine ranch is situated in the waters near the ecological city of Caofeidian District, Tangshan City. The main construction area is close to the coast, with convenient maritime transportation and well-developed communication infrastructure, which facilitates the breeding and cultivation of marine life and aids in the restoration of the natural marine environment. Considering factors such as the breeding season, fishing moratorium period, growth period, and wintering period, continuous surveys and the monitoring of benthic communities were conducted in the Caofeidian bottom-seeding marine ranch in September and November 2023 and January and May 2024. Six sampling stations were systematically arranged to ensure a uniform spatial distribution across the study area (Figure 1). Both water sampling and macro-benthic collection adhered strictly to the national standard, *Specifications for Oceanographic Survey—Part 6: Marine Biological Survey* (GB/T 12763.6-2007) [19]. In situ water quality parameters, including pH, conductivity, and dissolved oxygen, were measured using a portable multiparameter meter (SX836, Sanxin, Shanghai, China), while salinity was assessed with a salinity meter (ST20S, Ohaus, Shanghai, China). Measurements taken at the monitoring stations encompassed water temperature (WT), pH, dissolved oxygen (DO), and salinity (SAL). Additional water quality parameters were analyzed in the laboratory in accordance with standard procedures, such as those outlined in *Water and Wastewater Monitoring Methods* [20]. The seven water quality parameters assessed included chemical oxygen demand (COD), 5-day biochemical oxygen demand (BOD_5_), ammonia nitrogen (NH_4_^+^-N), nitrate nitrogen (NO_3_^−^-N), nitrite nitrogen (NO_2_^−^-N), soluble phosphate (PO_4_^3−^-P), and suspended particulate matter.

### 2.2. Sample Collection and Determination Methods

#### 2.2.1. Taxonomic Identification and Analysis

Benthic organisms were collected utilizing a grab sampler with a sampling area of 0.045 m^2^, with three replicate sediment samples obtained per station. The samples underwent sieving through a 0.50 mm mesh to eliminate impurities, followed by repeated washing into white porcelain trays for manual sorting. Each processed sample was independently verified by three researchers to ensure the validity of the dataset. The sorted specimens were preserved in a 5% formaldehyde solution for fixation. Taxonomic identification and morphological classification were performed using a stereomicroscope (Leica M205 A), after which biomass measurements were conducted. The analyzed specimens were subsequently returned to their original preservation vials and stored long term in a 10% formaldehyde solution, replacing the initial 5% fixative. The measured number of individuals and species wet weight were converted into habitat density (Density/m^2^) and Biomass (g/m^2^) according to the sampling area. Reference books for benthic biological taxonomy, such as *Catalogue of Marine Life of China, Illustrated*, provide a catalog of bivalves in China [21,22].

#### 2.2.2. Data Processing and Analysis

Dominant Species

The dominant species is judged from the following equation [23,24]:

*Y* = *n_i_*/*N* × *f_i_*

In the formula, *n_i_* represents the number of individuals of the i-th species, *N* denotes the total number of individuals, and *f_i_* signifies the frequency of occurrence of the *i*-th species. A species is classified as dominant when (*Y* > 0.02).

Bray–Curtis similarity analysis

The study employed hierarchical cluster analysis using PRIMER 5.2.8 software to assess similarity patterns in benthic community composition across various seasons [25,26]. Prior to the calculation of Bray–Curtis similarity coefficients, species abundance data were subjected to square root transformation [27]. These coefficients were subsequently organized into a similarity matrix for further community cluster analysis. The analysis was executed using IBM SPSS Statistics 27 software, wherein multidimensional scaling (MDS) was utilized to investigate temporal variations in benthic macroinvertebrate community composition.

Canonical correspondence analysis

The examination of species–environment relationships was performed using Canoco for Windows 4.5, adhering to standardized procedures for constrained ordination [28]. The results of the Detrended Correspondence Analysis (DCA) indicated a gradient length of greater than four standard deviations (SDs) for the first axis. Consequently, canonical correspondence analysis (CCA) was conducted to examine the multivariate effects of environmental factors on benthic communities, utilizing both species composition data and associated environmental variables. The CCA was performed using Canoco for Windows version 4.5, adhering to standardized protocols for constrained ordination.

### 2.3. Biodiversity

The Shannon–Wiener diversity index (*H′*) calculation formula [29] is expressed as the following equation:

$$H’=-\sum p_{i}\times {log}_{2}p_{i},$$

where $p_{i}$ is the proportion of the number of individuals of the *i*-th species at this station to the total number of individuals.

The Margalef richness index (*d*) calculation formula [30] is expressed as the following equation:

$$d=(S-1)/{log}_{2}N,$$

where *S* is the number of species in the community and *N* is the total number of individuals observed in the quadrat.

The Pielou evenness index (*J*′) calculation formula [31] is expressed as the following equation:

$$J’=H’\;/{log}_{2}S,$$

where *S* is the number of species in the community.

The Simpson diversity index (*D*) calculation formula [32] is expressed as the following equation:

$$D=1-\sum {p_{i}}^{2},$$

where $p_{i}$ is the proportion of the number of individuals of the *i*-th species at this station to the total number of individuals.

The diversity method uses the Student *t*-test + variance analysis, and *z*-score standardization is carried out before the analysis. The linear regression line adopts the most basic and simple linear regression analysis method to fit all test points into a straight line, and its fitting function equation is y = a + bx. In the above analysis, PRIMER 5.2.8 was used to calculate the Margalef species richness index (*d*), the Shannon—Wiener diversity index (*H′*), the Simpson dominance index (*D*) and the Pielou evenness index (*J′*) to measure and analyze the biodiversity of the large benthic animal community.

### 2.4. Correlation Analysis and Mantel Test Correlation 

After the large benthic animal communities were processed by log_10_ to make them more normally distributed, the average clustering method and Euclidean distance algorithm were used to conduct Pearson correlation analysis to explore the correlation between different large benthic animal communities and environmental factors [33]. A one-way ANOVA was used to analyze environmental factors with IBM SPSS Statistics 21 software to study the differences in environmental factors at different times, and *p* < 0.05 was used to indicate significant differences. In order to test the significance of environmental factors and the large benthic animal community, Mantel test correlation was used, and the Bonferroni method was used for p value correction. The analysis result of Mantel test heat-map plot was generated using the R 4.3.1 software package “vegan”. All the above analysis was carried out using the homogeneity test and normal distribution correction.

### 2.5. NMDS + PERMANOVA Analysis

The NMDS algorithm was used to study the similarity or difference in the composition of large benthic communities, and PERMANOVA (Adonis) was used for testing and identification. A nonparametric multivariate analysis of variance was used for sample distance (Bray–Curtis) [34].

## 3. Results

### 3.1. Species Composition and Distribution

**Figure 2:**
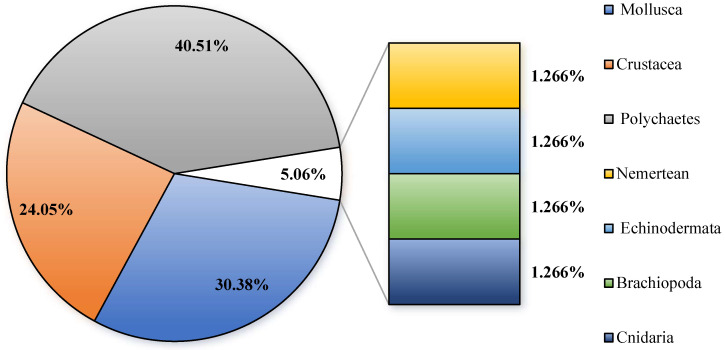
The species composition of macro-benthos in the study area.

The examination of macro-benthic communities across six stations within the bottom-seeding marine ranch in Caofeidian (Table 1 and Figure 2) revealed the collection of 79 macro-benthic species, of which 12 species remain unidentified at the species level. Polychaetes represent the most diverse group, comprising 32 species and accounting for 40.51% of the total species identified. Mollusks follow with 24 species, constituting 30.38%, while crustaceans are represented by 19 species, making up 24.05%. Additionally, single species of cnidarians, echinoderms, nemerteans, and brachiopods were identified, each contributing 1.27% to the total species count. Across four monitoring surveys, 16 species were consistently observed. *Ruditapes philippinarum* is the primary bottom-seeded species; however, only a limited number of individuals were encountered during the survey conducted in May.

The results of the analysis concerning the dominant macro-benthic species in the bottom-seeding marine ranch at Caofeidian (Table 2) indicate that *Musculus senhousei* consistently demonstrates absolute dominance across the four investigations, with *Scoloplos* sp. following in prevalence, except for January, and the other three sampling analyses showed that *Scoloplos* sp. was the dominant species.

**Figure 3:**
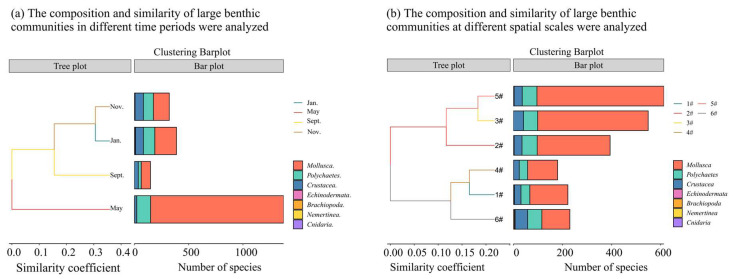
The composition and similarity of large benthic communities at different spatial scales were analyzed. Note: (**a**) Analysis of species composition and similarity of large benthic organisms under time change, (**b**) Analysis of species composition and similarity of large benthic organisms at spatial scale.

The species composition within the monitored area predominantly consists of mollusks, polychaetes, and crustaceans. As illustrated in Figure 3a, the seasonal variations in the macro-benthic community observed in this study are primarily reflected in changes in density rather than species composition. The Bray–Curtis similarity coefficient reveals that the greatest similarity is observed between the months of November and January. Temporally, the density in May is significantly higher than in the other three months, with *Musculus senhousei* exhibiting absolute dominance. Conversely, the species density in September is the lowest, potentially attributable to the adverse impact of fishing activities on the benthic habitat within this marine area.

As illustrated in Figure 3b, the temporal changes observed in the large benthic biota community in this study are primarily reflected in changes in density rather than species composition. The Bray–Curtis similarity coefficient indicates that the highest similarity is found between point 5 and 3. From the spatial distribution analysis, the density of point 5 is similar to that of point 3, and the assistance is higher than that of the other four sampling points. Conversely, site 4 has the lowest species density.

**Figure 4:**
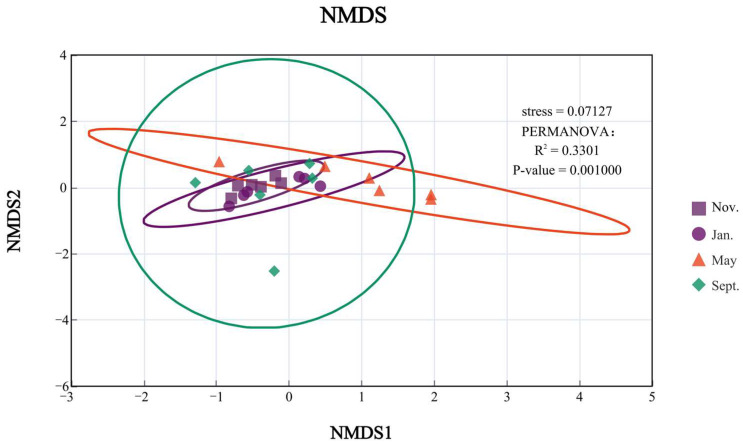
NMDS + PERMANOVA analysis of large benthic organisms in different months.

To investigate the clustering relationships of the four months, this study conducted NMDS analysis and cluster analysis using the Bray–Curtis distance matrix algorithm with a confidence interval of 0.95. As shown in the figure, the overall analysis indicates that November and January have the highest similarity with K, followed by May, while September has a lower similarity with other months. This may be due to the significant differences in the bottom-dwelling animal habitats caused by the fishing operations in September, which led to a greater variation, as shown in Figure 4.

**Figure 5:**
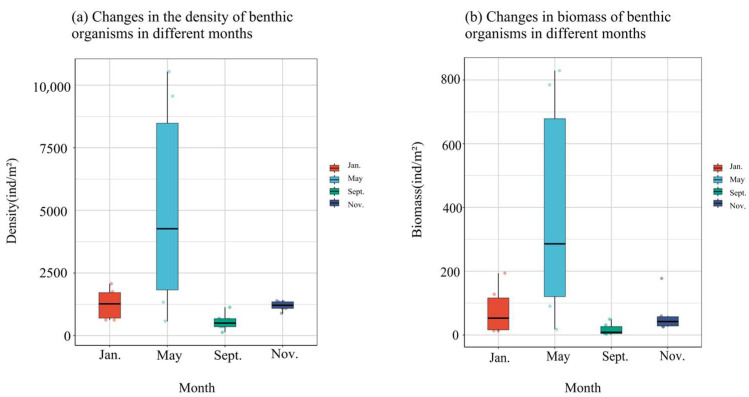
Density and biomass changes in large benthic animals in four months.

The temporal trends in the density and biomass of macro-benthos within the bottom-seeding marine ranch located in Caofeidian District, Tangshan City, exhibit a consistent pattern. Notably, both density and biomass peak in May, followed by a secondary peak in January, while reaching their nadir in September and November (Figure 5). Specifically, the density and biomass recorded in May are significantly elevated compared to other months. Conversely, September registers the lowest values for both parameters, with a marginal increase observed in November, although they remain relatively low.

**Figure 6:**
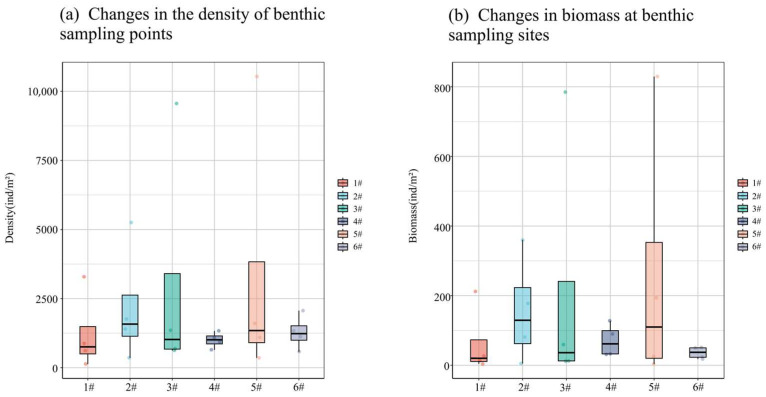
Density and biomass of large benthic communities with spatial variation.

The density and biomass of large benthic organisms, except for points 4 and 6, show a consistent trend with spatial changes. Notably, these indicators peak at point 5, followed by point 3, while they reach their lowest points at points 4 and 6 (Figure 6). Specifically, density and biomass at point 5 are significantly higher than in other months. Conversely, density is the lowest at point 4, and biomass is the lowest at point 6.

### 3.2. Analysis of Biodiversity

**Figure 7:**
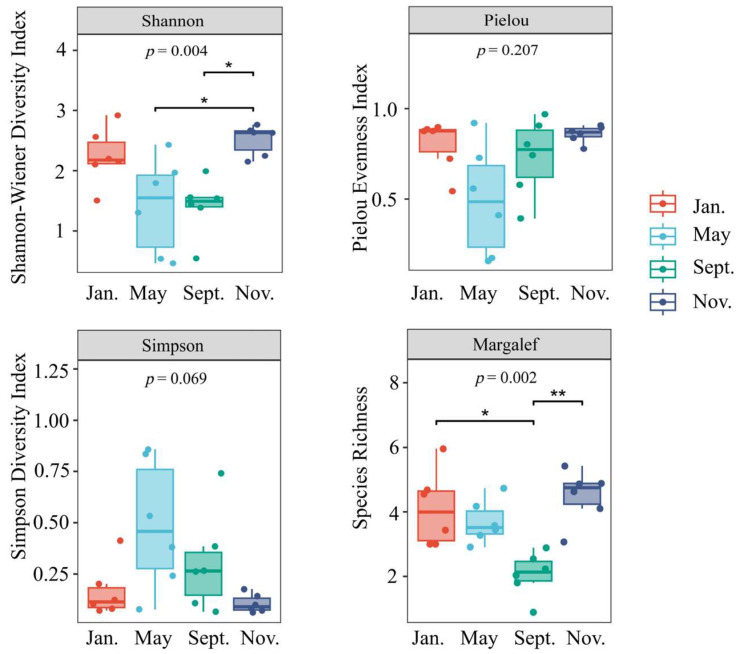
Box plots of macro-benthic alpha diversity indices in the study area. Note: Shannon diversity index (*H′*): Pielou uniformity index (*J*): Simpson advantage index (*D*): Margalef richness index (*d*); * a significant correlation at the 0.05 level, ** a significant correlation at the 0.01 level.

Figure 7 illustrates the biodiversity index of macro-benthos within the bottom-seeded marine ranch located in Caofeidian. The Shannon diversity index, with a significance level of *p* < 0.01, demonstrates relatively elevated values in November and January while exhibiting lower values in May and September. Notable differences in biodiversity are observed across these four months, potentially attributable to bottom trawling activities in the region. In contrast, the Pielou evenness index displays minimal variation. The *Simpson* index did not change significantly, with *p >* 0.05. Furthermore, the Margalef richness index, with a significance level of *p* < 0.01, is significantly reduced in September relative to the other months.

### 3.3. Analysis of Correlation with Environmental Factors

**Figure 8:**
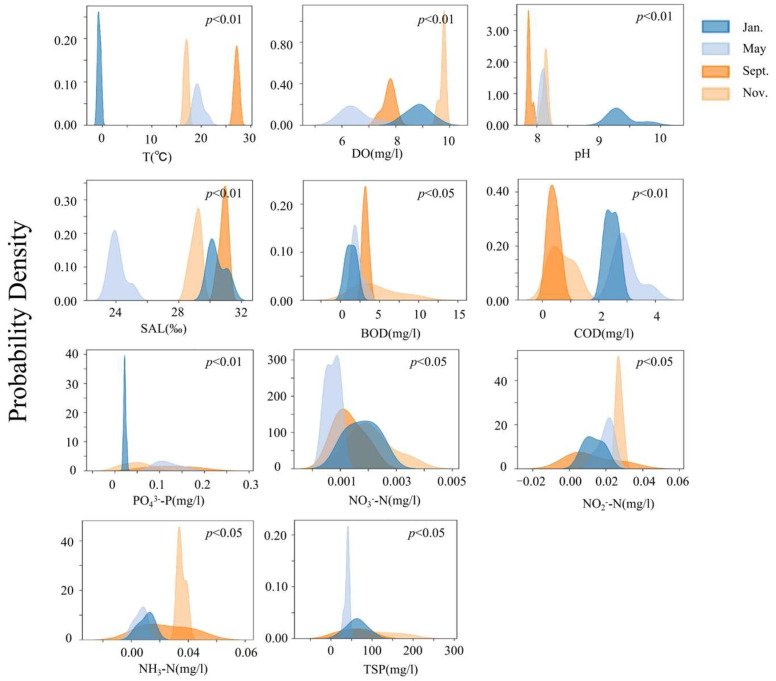
Analysis of seasonal variations in aquatic environmental parameters in the study area.

The temporal changes in the environmental factors in the bottom-seeding marine ranch in Caofeidian are shown in Figure 8. The physical and chemical indices of the water environment exhibit obvious characteristics among different survey months, and all the environmental factors show significant seasonal differences (*p* < 0.05). The chemical oxygen demand (COD) in May is significantly higher than that in other months. The biochemical oxygen demand (BOD) in November is significantly higher than that in other months (*p* < 0.05). The average values and variances of environmental factors in each month are shown in Table 3. The concentrations of dissolved oxygen, biochemical oxygen demand, nitrate, nitrite, ammonia nitrogen, suspended particulate matter and other ions in November all reach the highest values. The abundance of benthos in May is significantly higher than that in other months. Various ions in the water body are consumed in large quantities, resulting in relatively low levels of ionic indices. Due to the relatively low temperature in January, the concentrations of various ions and the environmental factors of the water body are all at average levels. The temperature is the highest in September, and the concentrations of various ions are at relatively low levels. In September, the temperature reaches its peak, while the concentrations of various ions remain relatively low.

**Figure 9:**
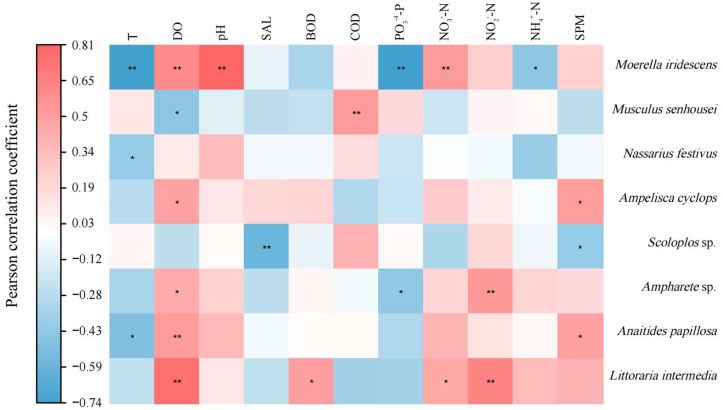
A Pearson correlation analysis of the relationship between dominant macro-benthic species and aquatic environmental factors in the study area. Note: * a significant correlation at the 0.05 level, ** a significant correlation at the 0.01 level.

A correlation analysis of the macro-benthic organisms in the bottom-seeding marine ranch in Caofeidian and the surrounding water environment (Figure 9) reveals that *Moerella iridescens* exhibits a significant positive correlation with dissolved oxygen (DO), pH, and nitrate nitrogen (NO_3_^−^-N) at the 0.01 significance level. Conversely, it shows a significant negative correlation with temperature, phosphate phosphorus (PO_4_^3−^-P), and ammonia nitrogen (NH_3_-N) at the same significance level. *Musculus senhousei* demonstrates a significant positive correlation with chemical oxygen demand (COD) at the 0.01 significance level and a negative correlation with DO at the 0.05 significance level. *Nassarius festivus* is negatively correlated with temperature. *Ampelisca cyclops* exhibits a positive correlation with dissolved oxygen (DO) and suspended particulate matter, both statistically significant at the 0.05 level. *Scoloplos* sp. demonstrates a significant negative correlation with salinity at the 0.01 level and a negative correlation with suspended particulate matter at the 0.05 level. *Ampharete* sp. shows a significant positive correlation with nitrite nitrogen (NO_2_^−^-N) at the 0.01 level, is correlated with DO at the 0.05 level, and exhibits a negative correlation with phosphate phosphorus (PO_4_^3−^-P) at the 0.05 level. *Anaitides papillosa* has a negative correlation with temperature at the 0.05 significance level, a significant positive correlation with DO at the 0.01 level, and a positive correlation with suspended particulate matter at the 0.05 level. *Littoraria intermedia* displays a significant positive correlation with both DO and NO_2_^−^-N at the 0.01 significance level and a positive correlation with biochemical oxygen demand (BOD) and nitrate nitrogen (NO_3_^−^-N) at the 0.05 level.

**Figure 10:**
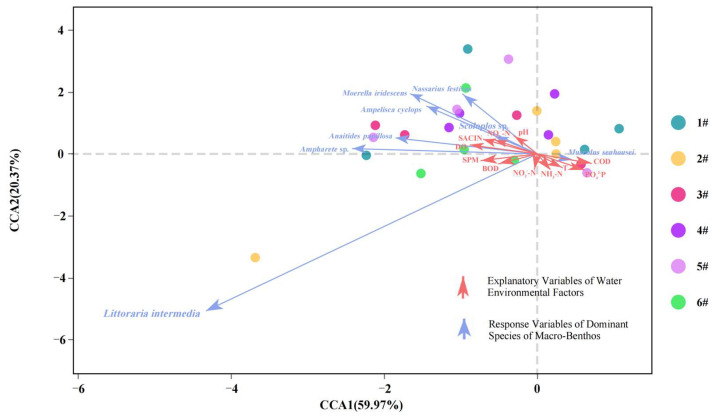
Canonical correspondence analysis (CCA) of benthic organisms and associated environmental variables. Note: In the legend, 1#–6# indicate sampling stations 1–6.

The detrended correspondence analysis (DCA) was conducted utilizing Canoco for Windows version 4.5. The analysis shows that the standard deviation (SD) value is higher than 3, prompting the use of canonical correspondence analysis (CCA) to assess the influence of environmental factors on macro-benthic communities. The results of this analysis are presented in Figure 10. The interpretation rate of CCA1 was 59.97%, that of CCA2 was 20.37%, and the cumulative interpretation rate of CCA1 and CCA2 was 80.34%. The distribution of each sampling point was scattered, and there was no aggregation trend. On the CCA1 axis, COD, PO_4_^−3^-P, NH_3_-N, and T were positively correlated, while the other environmental factors were negatively correlated; on the CCA2 axis, pH, SAL, DO, and NO_3_^−^-N were positively correlated, while the other environmental factors were negatively correlated.

**Figure 11:**
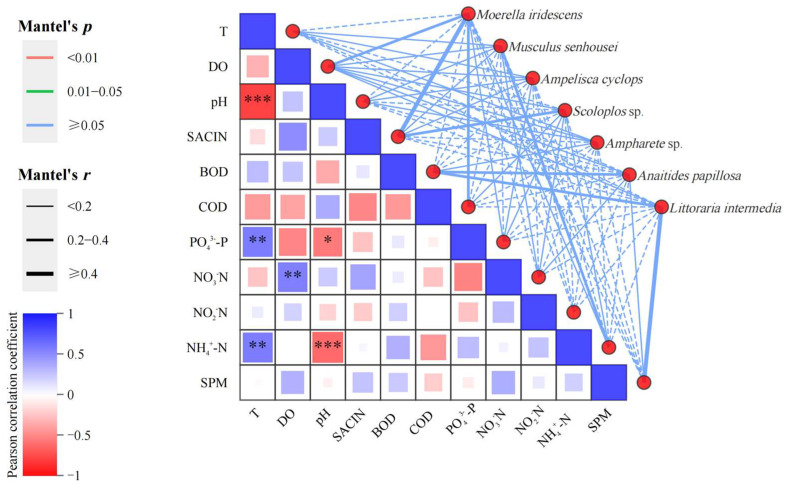
Large benthic organisms and environmental factors denoted on a Mantel test heat-map plot. Note: The width of the line indicates the magnitude of the absolute correlation (Mantel’s *r*), the color of the line indicates the range of the significance *p*-value (Mantel’s *p*), and the type of line (solid or dashed) indicates the sign of the correlation coefficient. * denotes *p* < 0.05, ** denotes *p* < 0.01, *** denotes *p* < 0.001, and no asterisk indicates *p* > 0.05.

The figure illustrates the significance of environmental factors and dominant large benthic communities. The network on the right uses the Mantel test to examine the correlation between each environmental factor and species. The width of the lines indicates the absolute value (Mantel’s *r*) of the correlation, the color of the lines indicates the range of the significance *p* value (Mantel’s *p*), and the line type (solid or dashed) indicates the sign of the correlation coefficient (Figure 11).

## 4. Discussion

### 4.1. Community Characteristics and Evolution of Dominant Species of Macro-Benthos

Under the dual pressures of environmental changes and human activities, the macro-benthic community maintains ecosystem stability via adjustments in species composition. Its dynamic changes can serve as an indicator of water health in marine ranches [35]. This study conducted the one-year monitoring of a bottom-seeded marine ranch in Caofeidian, using a combination of conventional grab sampling and trawl surveys to analyze the community structure of benthic animals and the action mechanisms of the water environment and human activities. The investigation findings revealed that 79 species of benthic animals were collected in this study, including 32 species of polychaetes, 24 species of molluscs, 19 species of crustaceans, and 1 species each of cnidarians, echinoderms, brachiopods and nemerteans. In terms of composition, it mainly comprises molluscs and annelids, similar to the findings of Quan Weimin et al. [36]. This composition is also consistent with the results of the 2022 settlement of macro-organisms on natural oyster reefs in the Caofeidian Sea area. However, the number of species in that research study is much less than that in this study. The reason may be the lower investigation frequencies and the analysis targeting only the macro-benthos settled on the natural oyster reefs, which leads to the differences in this study’s conclusions. The examination using the conventional grab sampler demonstrates that only a small number of bottom-seeded species, *Ruditapes philippinarum*, were collected. However, a certain number of bottom-seeded species (*Ruditapes philippinarum* and *Scapharca kagoshimensis*) were documented during the trawl survey. Meanwhile, a large number of *Rapana venosa* were also observed in the area.

This study shows that in the four months, the primary dominant species of the macro-benthos community in the bottom-seeding marine ranch in Caofeidian are primarily small bivalves and crabs and shrimps that live a buried–benthic life. In May, small bivalves become the dominant species. According to historical data records, the severe harm caused by *Musculus senhousei* occurs during the breeding period from April to May. This is due to their competition with shellfish for food, and its byssus connects into a sheet, making it challenging for other bivalves to attach to the surface, affecting the feeding, respiration and movement of cultured shellfish. In some cases, it even suffocates *Ruditapes philippinarum*, resulting in relatively small numbers of bottom-seeded species such as *Ruditapes philippinarum* and *Scapharca subcrenata*. This is consistent with the absolute dominant position of *Musculus senhousei* in May in this study [37,38]. Nonetheless, the biodiversity and numbers of bottom species gradually recovered in September and November. During the fishing ban period, the marine ranch resources are abundant, and the seabed is no longer continuously damaged during the fishing ban period. The impact of bottom species on dominant species *Musculus senhousei* has gradually diminished, and there is no long-term negative impact on bottom species. As a species with strong interference tolerance and pollution resistance, having a smooth surface and small individual size, *Musculus senhousei* remains the dominant species with the highest dominance level within four consecutive months, and its dominant position is quite significant. In this work, in the months when the abundance of *Musculus senhousei* in the bottom-seeded marine ranch of Caofeidian is high, the species richness is at a low level, and the number of individuals is also small. As the dominant species, *Musculus senhousei* seizes the ecological niche of bivalve shellfish, leading to a further decline in the overall biodiversity of benthic organisms. In parallel, it also affects the stability of the benthic animal community structure in the bottom-seeded marine ranch of Caofeidian. In September and November, its dominance level decreased to a certain extent. It is speculated that this change may be attributed to the fishing activities performed since September, and these activities may have disrupted the original structure of the benthic animal community. Among them, a large number of *Rapana venosa* were caught. With the considerable decrease in the number of *Rapana venosa*, the bivalve shellfish had fewer predators, and their numbers gradually recovered. The number of bottom-seeded species, such as *Ruditapes philippinarum* and *Scapharca kagoshimensis*, also relatively increased. A predatory relationship exists between the bottom-seeded species and *Rapana venosa*, and an ecological niche is occupied by *Musculus senhousei*. Under the impact of these dual factors, although the number of individual bottom-seeded species increases relatively, it remains at a low level. As the fishing activities gradually come to an end, the biodiversity in November begins to return to its original state gradually.

Among the macro-benthos in the bottom-seeding marine ranch of Caofeidian, *Musculus senhousei* is a species with strong interference tolerance and pollution resistance. Its reproductive quantity reaches its peak in May, occupying a dominant position. This finding is consistent with Cai Wenqian’s research results on the long-term variations in the dominant species in the macro-benthic animal community in Bohai Bay [39]. This phenomenon is consistent with the water body’s nutrient status and the frequent disturbances in the bottom-seeding marine ranch in Caofeidian, further confirming that the water body’s nutrient level in this area remains high and external disturbances are relatively frequent.

In recent years, the economy of marine ranches has displayed a rapid growth trend, yielding substantial profits from fishery resources. However, with the large-scale development of marine ranches, various challenges, such as illegal overfishing and the discharge of pollutants, have emerged. Consequently, the amount of pollutants entering the sea has significantly increased, notably raising the nutrient salt load in the sea area [40]. In addition, in the bottom-seeded marine ranch area, operations such as bottom trawling are frequently used to catch fish. This outcome has led to a decrease in the abundance, biomass and species diversity of macro-benthos in September and November, damaging the ecological functions of benthic organisms and weakening the self-purification capacity of the Bohai Sea marine ranch [41,42]. This trend will inevitably lead to a growing aggravation of water pollution [43,44]. It severely disrupts the habitats of benthic animals in the marine ranch [45]. This, in turn, results in significant changes in the community structure of macro-benthic animals across different months [36]. Currently, the most pressing issue for the ecosystem is addressing the damage to the seabed caused by trawling [46,47]. The bottom trawl exerted a strong destructive impact on the surface benthos. However, small bivalves that lead a buried–benthic lifestyle are less disturbed because of their living environment within the substrate. *Rapana venosa* primarily feeds on bivalve molluscs. In the surveyed area, *Rapana venosa* has an abundant food supply, reproduces rapidly and forms a large population. It is easily caught by trawls, generating a relatively small number of *Rapana venosa* after the fishing activities in September. In this benthic organism survey, *Rapana venosa* was only found in January, May and November and was not found in September. A predatory relationship exists between the bottom-seeded species *Ruditapes philippinarum*, *Scapharca kagoshimensis* and *Rapana venosa*. After the number of *Rapana venosa* decreased in September, the numbers of *Ruditapes philippinarum* and *Scapharca kagoshimensis* relatively increased. In this change, as a species tolerant to interference, *Musculus senhousei* has a smooth surface and small individual size. Some individuals can pass through the trawl’s mesh, resulting in a large population. They rapidly reproduce and take the dominant position in the marine ranch, thus having a substantial impact on the stability of the benthic animal community structure [48]. To mitigate the environmental effects of trawling, a trawl gear innovation trial supported by the European Union Fisheries Fund (EFF) has been successful in reducing both the amount of discarded catch and the impact of trawling on the seabed. The trial found that using a roller ball system instead of the traditional bottom trawling gear decreased the amount of discarded catch by 17%. With the roller ball system, the trawl moves forward by rolling along the seabed rather than scraping across it as with conventional trawls. This phenomenon reduces the trawl’s influence on the seabed and the accidental capture of non-target species, thereby enhancing the protection of marine ecosystems [49]. This invention may be used as the mainstream trawling method to protect bottom-sea-based marine ranches in the future, making a significant contribution to ecological protection.

### 4.2. The Diversity Characteristics of Macro-Benthic Animals

The *Shannon–Weaver* diversity index [50] is used to classify water quality levels for large benthic animals. The existing water quality classification standards of the diversity index in China are detailed as follows: *H* ≥ 3 clean; 2~3 light pollution; 1~2 moderate pollution; 0~1 heavy pollution; and *H* = 0 serious pollution [51]. However, the diversity index plays a crucial role in evaluating the stability of the macro-benthic animal community and the quality of the ecological environment [45]. In Chainho [52], the *H′* and *d* of the macro-benthic animal community were divided into five distinct grades (Table 4), including high, good, moderate, low and poor. The diversity index and richness index of macro-benthic animals in the bottom-seeded marine ranch of Caofeidian are at a moderate level. The specific value ranges are 0.45–2.9 for the diversity index and 0.89–5.42 for the richness index. The comparison of the degree of water pollution (Table 4) discovered that the health index of benthic organisms was always lower than the pollution index in each month. Generally speaking, there were significant differences in the types of pollution in Tangshan Caofeidian in different months. According to the *H′* diversity index, January, May, September and November were all *β*-Mesosaprobic. With *d* abundance, September was classified as *β*-Mesosaprobic, lightly polluted in May, and oligosaprobic in January and May (Table 5). The diversity index is at a low level in May, which is related to the reproductive period of *Musculus senhousei*, which reproduces in large numbers in May and seizes the ecological niche of other bivalve shellfish. This phenomenon results in species simplicity, which is consistent with the records in the book Marine Invertebrates in Northern China [36].

From the perspective of temporal distribution, the average value of *H′* in each month of the bottom-seeded marine ranch in Caofeidian shows that the average values in January and November are relatively high. In contrast, the average values in May and September are relatively low. Generally speaking, a higher index value often indicates better community stability and stronger adaptability to the ecological environment [53].

Regarding the phenomenon of the relatively low community diversity of macro-benthic animals in the marine ranch in May and September, the major reasons may be closely related to the eutrophication of the water body in this area, the reproductive period of *Musculus senhousei* and the fishing activities of the fishery resources. Villnäs and others highlighted that the long-term eutrophication of the water body would accelerate the renewal frequency of macro-benthic animal species, thereby triggering changes in community composition and a decrease in community functional diversity [54,55].

**Table 4:** The grading of the diversity and abundance indices of macro-benthic communities based on Chainho [52].

Classifications	Diversity Index (*H′*)	Richness index (*d*)
High	>4.0	>4.0
Good	3.0~4.0	>4.0
Moderate	2.0~3.0	2.5~4.0
Poor	1.0~2.0	<2.5
Bad	0.0~1.0	<2.5

**Table 5:** Relationship between large benthic biodiversity index and nutrient level and water pollution based on Zhang [56].

Types of Water Pollution	Diversity Index (*H′*)	Richness Index (*d*)
Oligosaprobic	>4.0	>4.0
Lightly polluted	>3.0~4.0	>3~4.0
β-Mesosaprobic	>1.0~3.0	2~3
α-Mesosaprobic to Polysaprobic	0~1	0~2

### 4.3. The Relationship Between the Density of Macro-Benthic Animals and Environmental Factors

As an essential component of the marine ecosystem, macro-benthic animals share a close relationship with various environmental factors at different temporal and spatial scales [57,58]. The impact of water environmental factors on macro-benthic animals is diverse. The influencing factors for the same species of macro-benthic animals in different spaces are different, and the various species of macro-benthic animals in the same space are affected to a dissimilar extent [59]. This work found that DO, pH, COD, NO_3_^−^-N, NO_2_^−^-N, PO_4_^3−^-P and NH_3_-N are the critical water environmental factors affecting the abundance variations in macro-benthic organisms in the bottom-seeded marine ranch of Caofeidian. According to the seawater quality standard (GB 3097-1997) [60], pH, DO and NH_3_-N all meet the Category I water quality standard in four months. COD in January and May were both in Category II water quality (Table 6). In September and November, the water quality was categorized as Category I. PO_4_^3−^-P meets Category II standards, except for the fact that May, January, September and November are all classified as Category IV standards. The abundance of benthic organisms is affected by various factors, including pH, DO and NO_2_-N, which showed a positive correlation. They had a negative correlation with temperature, salinity and PO_4_^3−^-P. Among them, biological species such as *Moerella iridescens*, *Ampelisca cyclops*, *Ampharete* sp., *Anaitides papillosa* and *Littoraria intermedia* were significantly positively correlated with the DO level; *Musculus senhousei*, the dominant species, depicted a negative correlation with DO, which is consistent with the features of *Musculus senhousei* in previous studies as a species with strong anti-interference tolerance, pollution resistance, and oxygen tolerance. Benthic species that are sensitive to oxygen and have weak tolerance may decrease or even disappear due to oxygen deficiency in a hypoxic environment [61]. The species with strong pollution resistance displayed a significant negative correlation with the dissolved oxygen content in water bodies and a significant positive correlation with the total phosphorus content. This outcome suggests that as the dissolved oxygen content in water bodies decreases and the nutrient content increases, the benthic animal community will gradually develop a community structure dominated by small individuals with strong pollution resistance. A long-term study conducted by Wen Shuke et al. [62] in Meiliang Bay, Taihu Lake, also demonstrated that an increase in the nutrient content in water often leads to the appearance of a high abundance of water silk worms, mosquito larvae and other pollution-tolerant species. Similarly, this is in good agreement with the conclusion of Wu Liang’s study [63] on the characteristics of benthic animal communities in the upper reaches of the Tiaoxi River in Lin′an. The conclusion of Cai Yongjiu et al. [64] is that oligochaetes have good adaptability to low-oxygen environments. The distribution of the apparent reverse species was significantly positively correlated with the dissolved oxygen content in water and significantly negatively correlated with the total phosphorus content. Wang Beixin et al. [65] and Wang Jianguo et al. [66] determined the pollution tolerance of benthic animals according to their occurrence probability in water bodies with different pollution levels. Among them, species such as hook shrimp, snails and mussels were slightly clean to moderately tolerant, which also revealed the response features of these groups to dissolved oxygen and total phosphorus. From 2016 to 2019, Jia Haibo examined the impact of seasonal hypoxia on large benthic animal communities in the Yangtze Estuary. Polychaetes exhibited a strong tolerance to hypoxia and were the dominant group in hypoxic areas. In contrast, the distribution trends of molluscs, crustaceans and echinoderms were opposite to those of polychaetes, with crustaceans showing weaker tolerance to hypoxia [67].

Nitrogen and phosphorus nutrients are essential for maintaining the health of ecosystems. However, excessive nutrient concentrations can accelerate the growth of blue-green algae, leading to water quality deterioration and a decrease in dissolved oxygen levels. In addition, these nutrients can increase the nutrient concentration in bottom sediments, directly affecting the survival of large benthic organisms, particularly in urban rivers with slower flow rates. Consequently, the community composition is dominated by pollution-tolerant species, generating poor biological integrity [68]. Along with the above-mentioned water environmental factors, the sediment environment, nutrients, interactions among organisms, and biological factors also substantially impact the distribution of macro-benthic animals, and they are important factors affecting the structure of biological communities [69,70]. In recent years, research on sediments and large benthic organisms in the Caofeidian area has also been conducted. In 2014, Liang Miao examined the pollution characteristics and bioaccumulation of heavy metals in the surface sediments and benthic organisms of Caofeidian. The heavy metal content in the surface sediments of the nearshore waters of Caofeidian fulfills the first-class standards for marine sediment quality. The order of heavy metals by concentration was Zn > Cr > Pb > Cu > As > Cd > Hg. The spatial distribution of heavy metals revealed a trend of high content near the Caofeidian reclamation area and low content far away from it [71]. However, there are few studies on the correlation between benthic organisms and water quality, as well as the design of water quality assessments. Due to previous sediment analyses conducted in this area, this study focused on the correlation between macro-benthic organisms and water environmental factors. Therefore, sediment is a limitation. Therefore, in-depth research on the crucial factors affecting the benthic animal community is considered of great significance for protecting regional biodiversity, evaluating the health status of marine ranches, and informing ecological governance and restoration efforts.

## 5. Conclusions

(1) Benthic organism surveys were carried out at six stations in the bottom-seeded marine ranch of Caofeidian each month, and a total of 79 species of benthic animals were captured. Among them, there were 31 polychaete species, 1 species each of cnidarians, echinoderms, and nemerteans, 19 crustacean species, and 25 mollusk species. The main dominant species in the macro-benthic animal community were mainly small bivalves and crabs and shrimps that lived a buried life. In May, small bivalves had become the absolute dominant species.

(2) The average value of *H′* for each month in the bottom-seeded marine ranch of Caofeidian shows that the average values in January and November are relatively high, while the average values in May and September are relatively low.

(3) The Pearson correlation analysis and CCA show that DO, pH, COD, NO_3_^−^-N, NO_2_^−^-N, PO_4_^3−^-P, and NH_3_-N are the key water environmental factors affecting the density changes in macro-benthic organisms in the bottom-seeded marine ranch of Caofeidian.

## Figures and Tables

**Table 1:** A taxonomic list of macro-benthos in the study area.

Phylum	Species	Jan.	May	Sep.	Nov.
Mollusca	*Eocylichna cylindrella*	+	+		+
	*Nassarius festivus*	+	+	+	+
	*Rapana venosa*	+	+		+
	*Nassarius succinctus*	+	+		+
	*Ringicula doliaris*	+	+	+	+
	*Neverita didyma*	+	+	+	+
	*Carssispira pseudopriciplis*		+		
	*Cerithidea sinensis*		+		
	*Eulima* sp.		+		
	*Mitrella burchardi*		+		
	*Retusa minima*		+		
	*Littorina undulata*	+			
	*Umbonium thomasi*	+			
	*Littoraria intermedia*				+
	*Terebra koreana*				+
	*Musculus senhousei*	+	+	+	+
	*Potamocorbula laevis*	+			+
	*Moerella iridescens*	+	+	+	+
	*Thyasira tokunagai*	+	+	+	
	*Ruditapes philippinarum* *		+		
	*Nucula tenuis*	+			
	*Philine kinglippini*	+			
	*Solen strictus*	+			
	*Theora fragilis*			+	
Crustacea	*Photis longicaudata*	+	+		+
	*Harpiniopsis vadiculus*	+			+
	*Ampelisca cyclops*	+	+	+	+
	*Pontocrates altamarimus*	+	+		
	*Ampelisca bocki*	+			+
	*Leptochela gracilis*		+		
	*Eriopisella sechellensis*	+			
	*Caviplaxus jiaozhouwanensis*				+
	*Paranthura japonica*	+	+	+	+
	*Eocuma lata*	+			+
	*Corophium* sp.	+	+	+	+
	*Diastylis tricincta*	+			
	*Iphinoe tenera*	+			
	*Cirolana japonensis*				+
	*Philyra acutidens*	+	+		
	*Pagurus* sp.	+			+
	*Nursia rhomboidalis*			+	
	*Paradorippe grannulata*				+
	*Balanus* sp.	+			
Polychaetes	*Maldanidae*	+	+		+
	*Mediomastus* sp.	+	+	+	+
	*Lumbrineris heteropoda*	+	+		
	*Marphysa sanguinea*	+	+		+
	*Amaeana occidentalis*	+	+	+	+
	*Onuphis geophiliformis*		+	+	
	*Ampharete* sp.	+	+		+
	*Nereidae*	+	+	+	+
	*Anaitides papillosa*	+	+	+	+
	*Amphictene japonica*	+	+	+	+
	*Sthenolepis japonica*	+	+		+
	*Pherusa* cf. *bengalensis*	+	+		+
	*Scoloplos* sp.	+	+	+	+
	*Potamilla acuminata*		+		+
	*Nephtys oligobranchia*	+	+	+	+
	*Tharyx multifilis*	+	+		+
	*Sternaspis scutata*	+	+	+	+
	*Gattyana pohailnsis*	+	+	+	
	*Diopatra chiliensis*		+		
	*Ophiodromus angustifrons*		+		
	*Aricidea fragilis*		+		
	*Capitella capitata*		+		
	*Sigambra bassi*		+		
	*Glycinde gurjanovae*	+			
	*Nephthys polybranchia*	+			
	*Paraprionospio pinnata*				
	*Notomastus latericeus*		+		
	*Spionidae*		+		
	*Glycera* sp.				+
	*Lumbrinereis* sp.				+
	*Magelona cincta*				+
	*Paralacydonia paradoxa*				+
Nemertean	*Nemertinea*	+			+
Echinodermata	*Ophiuroidea*	+	+		+
Brachiopoda	*Brachiopoda*	+			+
Cnidaria	*Anthopleura* sp.	+			

Note: The symbol “+” indicates the occurrence of the species; the symbol “*” represents the bottom-seeded species.

**Table 2:** Dominant species and dominance indices within macro-benthic communities in the investigated region.

Species	Jan.	May	Sep.	Nov.	Taxon
*Moerella iridescens*	0.11	/	/	0.12	Mollusca
*Musculus senhousei* *	0.32	0.70	0.39	0.15	Mollusca
*Nassarius festivus*	0.03	/	/	/	Mollusca
*Ampelisca cyclops*	0.07	/	0.18	/	Crustacea
*Scoloplos* sp.	/	0.02	0.02	0.03	Polychaetes
*Ampharete* sp.	/	/	/	0.03	Polychaetes
*Anaitides papillosa*	/	/	/	0.03	Polychaetes
*Littoraria intermedia*	/	/	/	0.11	Mollusca

Note: “/” indicates that the species is a non-dominant species during the sampling period, * means dominant species.

**Table 3:** Environmental factors of water body in study area (mean ± SD).

	Month	Jan.	May	Sep.	Nov.
Environmental Factor					
T (°C)		−0.65 ± 0.31	19.45 ± 0.99	27.16 ± 0.45	16.93 ± 0.39
DO (mg/L)		8.84 ± 0.43	6.49 ± 0.53	7.74 ± 0.21	9.75 ± 0.10
pH		9.36 ± 0.21	8.09 ± 0.04	7.88 ± 0.03	8.13 ± 0.03
SAL (‰)		30.11 ± 0.01	24.10 ± 0.50	30.87 ± 0.26	29.08 ± 0.32
BOD (mg/L)		1.36 ± 0.64	2.10 ± 0.65	3.20 ± 0.35	4.63 ± 2.75
COD (mg/L)		2.45 ± 0.22	2.97 ± 0.43	0.39 ± 0.19	0.69 ± 0.41
PO_4_^3−^-P (mg/L)		(2.20 ± 0.77) × 10^−2^	0.12 ± 0.03	0.12 ± 0.04	0.05 ± 0.03
NO^3−^-N (mg/L)		(0.17 ± 0.06) × 10^−2^	(0.72 ± 0.07) × 10^−3^	(0.13 ± 0.05) × 10^−2^	(0.21 ± 0.07) × 10^−2^
NO^2−^-N (mg/L)		(1.30 ± 0.53) × 10^−2^	(1.90 ± 0.41) × 10^−2^	0.01 ± 0.01	(2.70 ± 0.17) × 10^−2^
NH_3_-N (mg/L)		0.01 ± 0.01	(0.60 ± 0.35) × 10^−2^	0.02 ± 0.02	(3.50 ± 0.28) × 10^−2^
TSP (mg/L)		62.60 ± 23.58	39.62 ± 4.90	56.62 ± 39.81	101.66 ± 54.95

**Table 6:** Seawater water quality standard (GB 3097-1997) [60].

Environmental Factor	Category I	Category II	Category III	Category IV
pH	7.8~8.5	7.8~8.5	6.8~8.8	6.8~8.8
DO	>6	>5	>4	>3
COD	≤2	≤3	≤4	≤5
Inorganic nitrogen	≤0.20	≤0.30	≤0.40	≤0.50
Iabile phosphate	≤0.015	≤0.030	≤0.030	≤0.045

## Data Availability

The data supporting this study’s findings are available from the corresponding authors upon reasonable request.
